# Lipid-based surface engineering of PLGA nanoparticles for drug and gene delivery applications

**DOI:** 10.1186/s40824-016-0081-3

**Published:** 2016-10-31

**Authors:** Rajendran JC Bose, Soo-Hong Lee, Hansoo Park

**Affiliations:** 1 School of Integrative Engineering, Chung-Ang University, Seoul, Republic of Korea; 2 Department of Biomedical Science, CHA University, Seongnam-si, Gyeonggi-do Republic of Korea

**Keywords:** Surface engineering, Lipids, PLGA nanoparticle, Self assembly, Cell membrane derived lipid vesicles, Biomimetic fucntionalization, Controlled drug release, Gene delivery

## Abstract

The use of poly(lactic-co-glycolic acid) (PLGA)-based nanocarriers presents several major challenges, including their synthetic hydrophobic surface, low transfection efficiency, short circulation half-life, and nonspecific tissue distribution. Numerous engineering strategies have been employed to overcome these problems, with lipid-based surface functionalization of PLGA nanoparticles (NPs) showing promising results in the development of PLGA-based clinical nanomedicines. Surface engineering with different lipids enhances the target specificity of the carrier and improves its physicochemical properties as well as NP-cell associations, such as cellular membrane permeability, immune responses, and long circulation half-life in vivo. This review focuses on recent advances in the lipid-based surface engineering of PLGA NPs for drug and gene delivery applications.

## Background

Nanotechnology has been widely studied to improve the pharmacokinetics and therapeutic efficacy of a myriad of drugs, including proteins, genes, and other small molecules [1–4]. In recent years, several therapeutics based on poly(lactic-co-glycolic acid) (PLGA) nanoparticles (NPs) (hereinafter abbreviated PNPs) have entered into preclinical development or are being investigated in biomedical research, owing to their attractive properties of biodegradability, biocompatibility, ease of processing, and sustained release [5–8]. To optimize their clinical potential, considerable efforts have been devoted to understanding the mechanism of interaction between the PNP surface and its biological environment [9]. The major barrier to the use of the PNP is its hydrophobic surface, which is often recognized as foreign material by immune cells, leading to its rapid elimination from systemic circulation [10]. In addition, this surface property of the PNP limits its cellular membrane permeability, often resulting in poor transfection efficiency in in vitro experiments [11]. To address these limitations, numerous strategies have been investigated [9–14], among which lipid-based surface engineering has been shown to be effective in preclinical studies owing to the biomimetic and biocompatible advantages of this strategy [10, 12, 15]. Currently, a broad range of lipids have been determined to significantly improve the therapeutic potential of the PNP platform [13, 16, 17]. The present review focuses on recent advances in the lipid-based surface engineering of PNPs for drug and gene delivery applications. We provide recent information regarding the surface engineering methods based on synthetic lipids and on natural cell-membrane-derived lipid vesicles (nanoghosts) [11, 15, 18, 19]. The methods used in lipid-based surface engineering, and the properties and biomedical applications of the lipid-PLGA hybrid nanoparticles (LPHNPs) produced, are described in detail. Discussion of other types of surface modification techniques is limited as these are not within the scope of this review.

## Lipid-based surface engineering of PLGA nanoparticles

Lipids are hydrophobic or amphiphilic molecules, present in various molecular types such as fatty acids, oils, steroids and waxes [20]. Among all, glycerophospholipids are the main component of biological membranes, which composed of a glycerol molecule linked to a phosphate group (PO_4_^2−^) and to two fatty acids [20]. These phospholipids have been widely employed for the surface engineering of PNPs. Phospholipids such as phosphatidylcholine, phosphatidylinositol, phosphatidylglycerol, phosphatidylethanolamine, phosphatidylserine, and phosphatidic acid are less stable in nature [21, 22]. Thus their synthetic counterparts have been synthesized by modification of the nonpolar and polar regions of the phospholipid molecules [21]. Differentially charged synthetic phospholipids, such as zwitterionic, cationic, anionic, and neutral phospholipids (e.g., DOTAP, and sterol lipids such as cholesterol), are often used in biomedical engineering [13, 15]. Polyethylene glycol (PEG) is a hydrophilic lipid that has been largely applied to improve the circulation half-life of NPs in blood [17, 18, 23, 24]. The amphiphilic nature of phospholipids allows them to form organized structures, such as vesicles or membranes, when immersed in an aqueous environment. Additionally, lipid self-assembly on the polymeric substrate depends on their surface properties, such as charge and nature of substrate (hydrophilic/hydrophobic) [16]. In general, electrostatic attraction and hydrophobic interactions are the major chemical forces responsible for the lipid self-assembly process on PNP surfaces [17, 18]. The incorporation of anionic or cationic lipids into a phospholipid bilayer yields charged vesicles that can be adsorbed onto oppositely charged polymeric NPs via electrostatic attraction [13]. Neutral phospholipids, such as phosphatidylcholine and dipalmitoylphosphatidylcholine, adsorb and self-assemble onto hydrophobic polymeric surfaces through hydrophobic interactions in order to reduce the free energy of the system [15, 18]. The hydrophobic tails of lipids adsorb onto the hydrophobic PNP surface, while the hydrophilic head groups of the lipids extend into the external aqueous environment, forming a lipid-monolayer-coated PNP imparting aqueous stability [15]. As more and more lipids are added to the NP dispersion, vesicles form in addition to lipid-monolayer-coated NPs [17, 18]. The latter can interact with the vesicles via van der Waals interactions, resulting in further lipid deposition and thus increasingly larger numbers of lipid monolayers onto the PNPs [18].

Advantages in using synthetic lipids, such as DOTAP, for surface engineering of PNPs include the ease of processing and customization [13, 16]. However, adverse effects, such as the cytotoxicity of the PNPs produced and the immune responses they elicit, have fueled new surface engineering strategies, such as natural cell-membrane-derived lipid vesicles (nanoghosts) [10, 12, 19, 25, 26]. The motivation behind this new development lies in the fact that natural cell membrane components (i.e., lipids, proteins, and carbohydrates) have complex structures that are difficult to mimic with synthetic lipids alone [19]. Researchers have investigated various natural cell-membrane-derived nanoghosts from erythrocytes (RBCs), leukocytes, platelets, stem cells, and cancer cells for the surface engineering of PNPs [10, 12, 14, 19, 25, 26]. This cell membrane functionalized nanoparticles (CMFNPs) have the combined advantages of both cell mimetic surface and polymeric NPs [26, 27]. The lipid and protein compositions of these nanoghosts offer the unique advantage of source cell surface to the PNPs [27]. For instance, RBC-derived nanoghosts enable the PNPs to have extended the circulation half-life in vivo [10].

## Methods in lipid-based surface engineering of PLGA nanoparticles

**Fig. 1:**
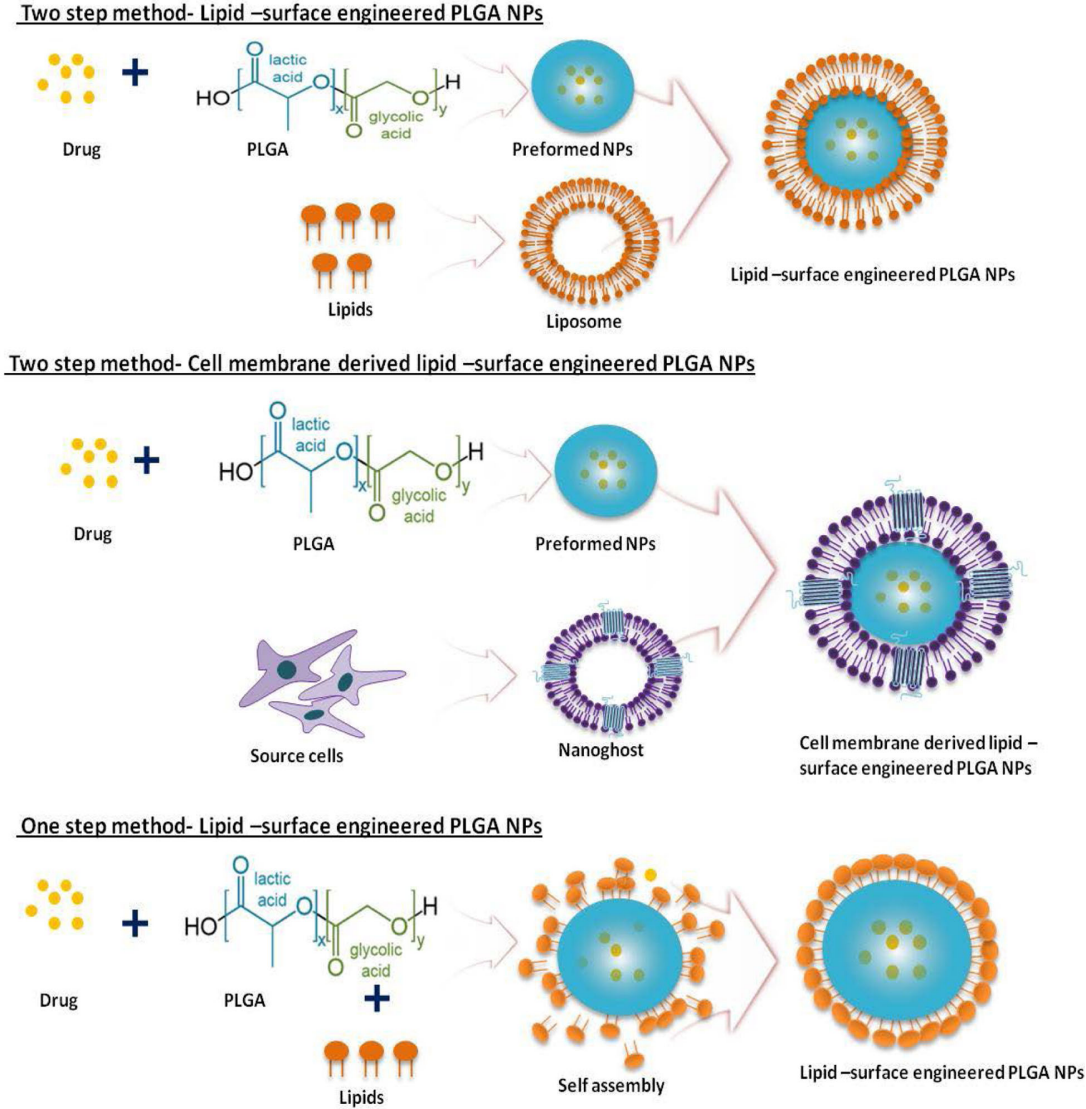
Schematic diagram explains the different methods in the lipids based surface engineering on PLGA nanoparticles

There are many methods employed for the lipid-based surface engineering of PNPs [17, 19, 28, 29]. Fig. 1 depicts different methods for fabrication of lipid-PNPs. Fabrication using synthetic lipids can be achieved by either the classical two-step method or the contemporary single-step process [17, 18]. The selection of right method of preparation is depends on various factors such as size, shape and nature of drug incorporation with the engineered nanoparticles. In the classical two-step method, the preformed PNPs are mixed with preformed lipid vesicles, where the latter adsorb onto the polymeric NPs by electrostatic interactions [30]. Non-conventional soft lithography and spray drying methods are also applied to create PNPs of different sizes and shapes [31]. Top-down methods have been generally employed for the nanoghost-based surface engineering of PNPs, the major steps of which (including separation of cell membranes and surface engineering methods) have been briefly discussed in our recent review [19].

The nanoprecipitation and emulsification-solvent-evaporation (ESE) techniques can be customized for PNP preparation by the single-step method [13, 15, 16, 32]. In the single-step nanoprecipitation method, where the PLGA polymer and the lipids are either dissolved in organic solvent or the lipid and/or the lipid-PEG are dispersed in water. The PLGA polymer solution is then dropped into an aqueous phase under constant stirring, causing the precipitation of PNPs [13, 16]. During solvent evaporation, the lipids self-assemble around the PNP core via hydrophobic interactions, with their hydrophobic tails attached to the core and their hydrophilic heads facing the external aqueous surrounding, resulting in the formation of LPHNP structures [13, 15, 16, 33]. The ESE method is distinguished into single and double emulsification processes. In the single ESE method, the PLGA polymeric solution is added to an aqueous phase containing the lipids to form an oil-in-water emulsion [17, 34]. In the double ESE method, the organic phase containing the PLGA polymer solution and lipids is emulsified with an aqueous buffer, and the resultant water-in-oil emulsion is further emulsified with a stabilizer or lipid-PEG to form a water-in-oil-in-water emulsion [15, 17]. In both methods, once the organic solvent has evaporated, the PNP core is formed, around which the lipids will subsequently self-assemble, similar to the nanoprecipitation method [15, 18].

**Fig. 2:**
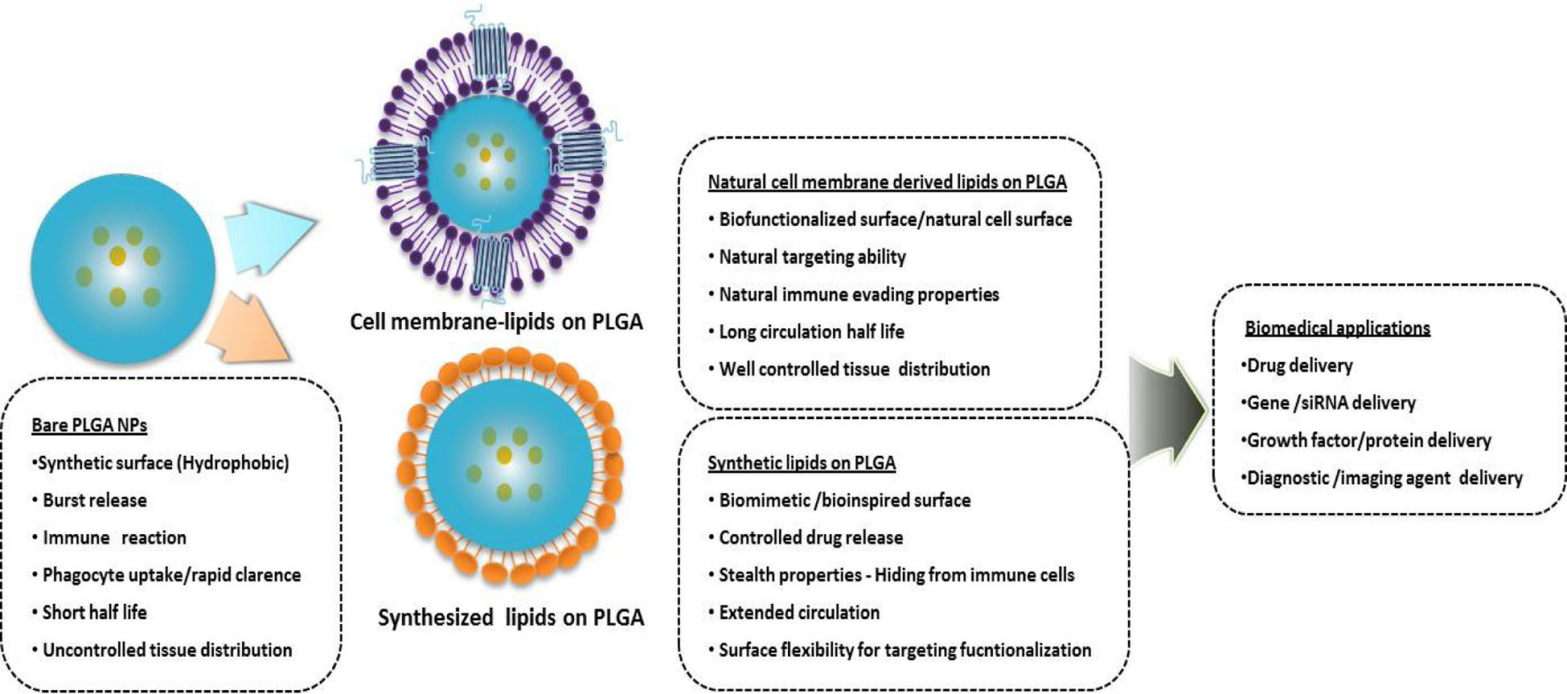
Advantage and application of different lipids-surface engineering on PLGA nanoparticles. The bare PLGA nanoparticles have major drawbacks such as hydrophobic surface, rapid phagocytic clearance and initial burst release. Cell membrane derived lipid vesicles or synthetic lipids based surface engineering on PLGA nanoparticles prevent the fore told drawbacks and improve the clinical performance

Selection of the correct lipid-based surface engineering method to use is crucial and depends on the nature of the lipid-PLGA surface chemistry for the desired biomedical applications [17, 18, 35]. For instance, we employed the single-step ESE method for gene delivery applications, and the nanoprecipitation method for the encapsulation and sustained release of antiproliferative agents [13, 15]. In contrast, nanoghost-based surface engineering cannot be achieved through single-step methods, requiring a conventional two-step top-down approach instead [10, 12, 25, 26]. A list of LPHNP studies that employ the two-step and single-step methods are provided in Table 1. Because of the perceived advantages of LPHNPs over other existing hybrid systems, significant effort has been made to understand their basic structure [36]. LPHNPs commonly comprise a hydrophobic PNP core, a lipid monolayer or bilayer surrounding the core, and a lipid-PEG corona [18]. Fig. 2 depicts the advantage of different lipid surface engineering on PNPs.

**Table 1:** List of Lipid-PLGA nanoparticles and applications

Lipid	Polymeric core	Preparation method	Encapsulated Drug	Size(nm)	Charge(mV)	Application	Reference
Lecithin/DSPE-PEG	PLGA	One step	Au/QDs	50–60	NA	Diagnostic/Optical imaging	[32]
Chol			Curcumin	203	-0.67 ± 1.23	Cancer therapy	[34]
EPC, DOPE-Tf/TPGS			Aromatease Inhibitor	150–190	-18 ± 2	Cancer therapy	[56]
Lecithin, DMPE,DTPA/DSPE-PEG			Docetaxel, ^90^Y,^111^In	65	35	Cancer therapy	[57]
Lecithin/DSPE-PEG			Paclitaxel - Gemcitabine	70 ± 1	-51 ± 2	Cancer therapy	[58]
Lecithin/DSPE-PEG			Paclitaxel	55 ± 1	NA	Treatment of coronary artery disease	[59]
DSPE-PEG			docetaxel	25	-10	Cancer therapy	[60]
EPC DSPE-PEG		Two step	siRNA	225 ± 8	- 10	Gene Delivery	[61]
FA-QLCS,Chol, PEG-OQLS			Paclitaxel	184 ± 7	22 ± 4	Targeted nanoparticle for cancer therapy	[62]
Chol, DOTAP, DSPE-PEG			BSA	~144	+4–6	Vaccine delivery	[63]
DOPC,DOTAP, DSPE-PEG		One step	Sirolimus	174 ± 9.6	−14 ± 0.3	Sustained antiproliferative therapy for the treatment of restenosis	[13]
			Propolis	183 ± 13.7	−21 ± 2.08		
DOTAP			Sirolimus	201 ± 10.3	36 ± 6.5		
			Propolis	205 ± 9.4	14 ± 4.1		
DOPC			Sirolimus	201 ± 8.8	−24 ± 1.2		
			Propolis	212 ± 11	−32 ± 2.5		
DOPC,DOTAP			Sirolimus	186 ± 8.6	−12 ± 3.5		
			Propolis	194 ± 11.7	−19 ± 2.4		
DOTAP (6 %)		One step	pDNA	209 ± 10.4	36 ± 5.4	Gene delivery	[15]
DOTAP (12 %)				186 ± 7.9	49 ± 2.3		
DOTAP (18 %)				163 ± 5.6	57 ± 2.5		
DOTAP (24 %)				154 ± 5.2	64 ± 3.2		
RBC membranes		Two step	Doxorubicin	70–90	-10 ± 2.7	Long circulating carrier	[44]
Platelet membranes			Docetaxel	113.4 ± 1.2	-29.5 ± 1.2	Natural targeted drug delivery. Long circulating carrier	[26]
			Vancomycin	200.3 ± 3.1	-30.2 ± 1.0		
Cancer cell membranes			NA	~110	-35 ± 5	Natural cancer targeted drug delivery. Whole cell cancer vaccine	[64]
Monocyte membranes			Doxorubicin	~150–180	−16.5	Natural cancer targeted drug delivery	[14]
Adipose derived Stem cell membranes			VEGF	100–150	-15 ± 1.3	Natural targeted drug delivery. Stem cell mimetic nanocarrier	[12]

## Applications of lipid-based surface-engineered PLGA nanoparticles

### Drug delivery

**Fig. 3:**
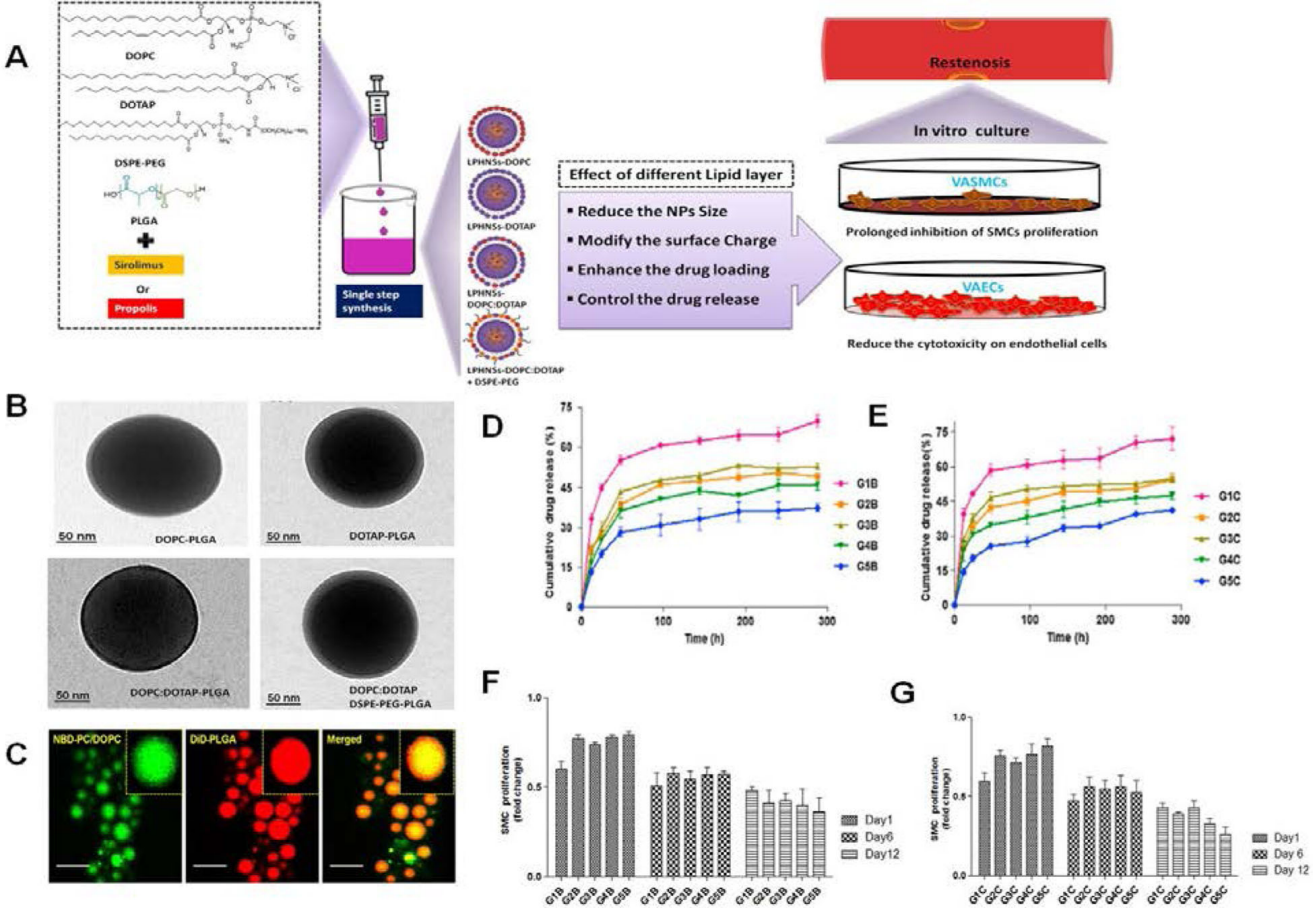
Effect of different lipids on properties of lipid-PLGA nanoparticles (LPHNPs) for drug delivery application. **a** Schematic diagram for LPHNPs preparation and in vitro characterization, **b** TEM-image of different lipids-PLGA NPs, **c**CLSM image of LPHNPs (Core-Shell structure), **d** Effect of different lipids on sirolimus release kinetics from LPHNPs, **e** Effect of different lipids on propolis release kinetics from LPHNPs, **f** Inhibitory effect of Sirolimus-LPHNSs on HASMCs proliferation, **g** Inhibitory effect of Propolis-LPHNSs on HASMCs proliferation. (Reproduced from reference.13)

The different surface engineering methods available for producing LPHNPs have enabled the incorporation of an extensive range of drugs, regardless of their properties (e.g., water solubility and ionicity) [17, 37, 38]. LPHNPs have been demonstrated as a promising drug delivery platform owing to their greater biomimetic and biodegradable abilities, with customized targeting features [17, 39]. The selection of lipids allows for further customization of PNPs with desired drug delivery features, such intracellular drug delivery or extended circulation with target-specific localization [15, 31]. The lipids on the PNP surface can act as a biocompatible fence to control the drug-releasing kinetics and improve the therapeutic efficacy of the drug [13]. Recently, our research group applied this strategy to improve the antiproliferative efficacy of a drug in an in vitro system, where the effects of a higher drug concentration and of the synthetic polymeric surface of the drug-eluting stent were reported for stent-associated thrombosis [40]. Fig. 3 show the effect of different lipids on properties of lipid-PLGA nanoparticles (LPHNPs) for sirolimus or propolis delivery. In Fig. 3, the schematic diagram show the method of preparation of drug encapsulated LPHNPs and its characterization including the morphology, release kinetics and inhibitory effect of sirolimus or propolis -LPHNSs on HASMCs proliferation. We demonstrated that different lipid-based surface engineering techniques used to produce sirolimus/propolis-loaded PNPs significantly improved the antiproliferative efficacy of the drugs against smooth muscle cells, while reducing the drug-mediated cytotoxicity on endothelial cells.

Classically, PEG lipids have been employed to improve the biodistribution and circulation half-life of PNPs, as the PEG corona provides steric stabilization and acts as a protective layer [41, 42]. Recently, however, PNPs engineered with RBC-membrane-derived nanoghosts were demonstrated to be better biomimetic long-circulating nanocarriers than PEG-based PNPs [10, 43]. Moreover, Aryal et al. demonstrated that the RBC nanoghosts on the PNP surface could act as a diffusion barrier to better control the drug release, as compared with PEG-based PNPs, thereby enhancing the therapeutic efficacy of the drug in acute myeloid leukemia cells [44].

Bare PNPs have the major drawback of nonspecific target localization, resulting in uncontrolled tissue distribution of the drug. To improve the PNPs’ site-specific localization, various strategies have been employed [10]. Lipid-based surface engineering allows for target functionalization of PNPs through either conjugation chemistry or by conferring upon them cell-membrane-mediated natural targeting capabilities [26, 45, 46]. Because target functionalization by various conjugation chemistries has already been reviewed by several authors, we focus here instead on the cell-membrane-mediated approach.

Cell-membrane-derived nanoghosts have the combined advantages of synthetic and biological features for improved target specificity and drug efficacy [14, 19]. Thus, nanoghost-based surface engineering has been actively used to target PNPs to the diseased sites [10, 14, 19]. For instance, Fang et al. demonstrated a significant enhancement of the natural binding ability of drug-loaded PNPs to the source cancer cells owing to the presence of adhesion molecules [46, 47]. Correspondingly, platelet cells have a natural ability to home in on injured blood vessels as well as circulating pathogens. Thus, surface engineering of PNPs with platelet-membrane-derived nanoghosts provides them with the natural platelet-like targeting functions [26, 48]. Krishnamurthy et al. demonstrated that the monocyte-membrane-derived nanoghost-based surface engineering of DOX-loaded PNPs resulted in higher cytotoxicity in MCF-7 breast cancer cells [14, 19, 49]. In addition, the multicompartmental nature of LPHNPs has an advantage, in that multiple therapeutic agents can be incorporated into the different compartments of the NPs [17, 18, 50]. Altogether, LPHNPs have been employed mostly for numerous drug delivery applications. The list is quite extensive and we therefore provide only the most recent applications in Table 1.

## Gene delivery

Although traditional nonviral delivery systems such as liposomes and polyethylenimine (PEI) have been confirmed to be effective in in vitro and in vivo models, their clinical potential is drastically limited because of their instability and higher cytotoxicity upon systemic administration [51]. LPHNPs have emerged as a novel nonviral gene delivery system owing to their improved stability and low cytotoxicity profiles. In particular, cationic lipid (DOTAP)-layered PNPs have numerous advantages over lipoplex (liposome)-, polyplex (PEI)-, and viral-based vectors, such as their large-DNA incorporation ability, higher transfection efficiency with mild cytotoxicity, and feasibility for pilot-scale production [18, 52].

**Fig. 4:**
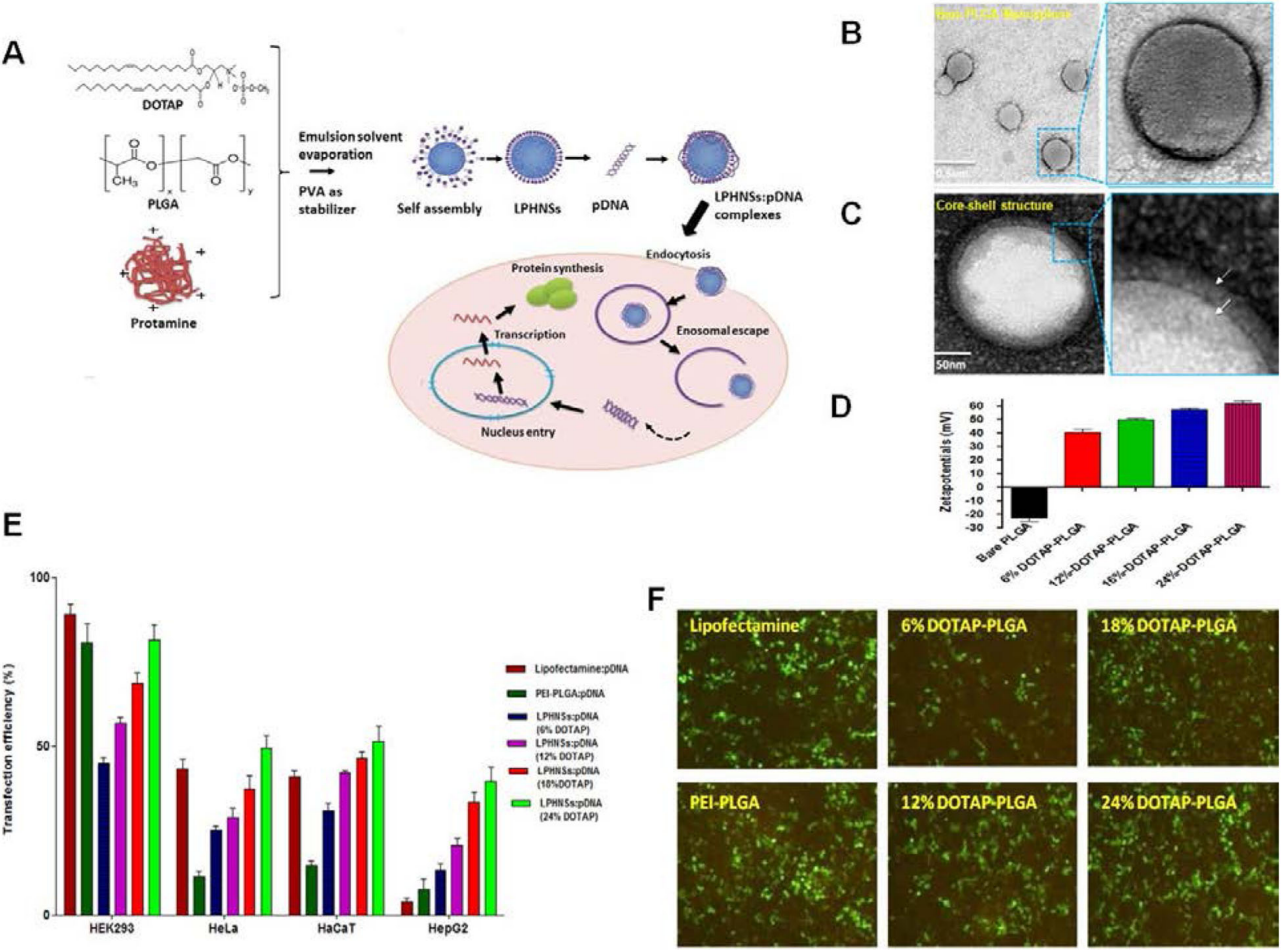
Effect of DOTAP lipid on properties of lipid-PLGA nanoparticles (LPHNPs) for gene delivery application. **a** Schematic diagram for LPHNPs-DNA complex formation and transfection, **b** TEM-image of bare PLGA NPs, **c** TEM-image of LPHNPs, **d** Effect of DOTAP concentration on LPHNPs surface charge, **e** Effect of DOTAP concentration on LPHNPs transfection efficiency in various cells (Flow cytometry analysis) and **f** fluorescence microscopy. (Reproduced from reference.15)

As illustrated in Fig. 4, we investigated the effect of DOTAP lipid on properties of lipid-PLGA nanoparticles (LPHNPs) for gene delivery application. We demonstrated the strong influence of DOTAP concentration on the surface properties of the LPHNPs, impacting their plasmid DNA-binding capacity, cytotoxicity, and transfection efficiency in HeLa, HaCaT, HEK293T, and HepG2 cells [11, 15]. However, the use of LPHNPs to reduce nonspecific protein binding has not been well investigated. At present, studies on cationic LPHNP–DNA complexes are still in their infancy, and most investigations have focused only on nanocarrier preparation and characterization.

## Conclusions

Lipid-based surface engineering of PNPs offers several advantages in the development of drug and gene delivery platforms, including a broad range of flexible strategies and ease of surface engineering, as well as extended circulation half-life, better target specificity, reduced cytotoxicity, and improved transfection efficiency of the nanocarriers [13, 36, 53]. Collectively, all of these features make the lipid-based surface engineering strategies ideal for improving the clinical performance of PNPs [18, 24]. Although enormous progress has been made in the area of nanoengineering, many challenges remain that potentially hinder the translation of PNPs to the clinical arena [5, 54]. Lipid-based surface engineering can be further optimized to improve the clinical outcomes of PNPs in drug and gene delivery applications [55]. Precise control of the surface engineering with different lipids as well as of their concentration on PNPs is critical in gene delivery, as these factors directly influence the efficiency of nanocarriers [15, 51].

## Abbreviations

DOPC: 1, 2-dioleoyl-sn-glycero-3-phosphocholine ESE, emulsification–solvent–evaporation
DOTAP: 1, 2-dioleoyl-3-(trimethyl ammonium) propane
DOX: Doxorubicin
DSPE-PEG: 1, 2-distearoyl-sn-glycero-3-phosphoethanolamineN-[methoxy-(polyethyleneglycol)-2000] (ammonium salt)
HaCaT: Human keratinocyte cell Line
HEK293T: Human embryonic kidney 293 cells
HeLa: Human cervical cancer cell line
HepG2: Human hepatocellular carcinoma cell line
LPHNPs: Lipid–PLGA hybrid nanoparticles
pDNA: Plasmid deoxyribonucleic acid
PLGA: Poly(lactic-co-glycolic acid)
